# The influence of music performance anxiety on career expectations of early musical career students: self-efficacy as a moderator

**DOI:** 10.3389/fpsyg.2024.1411944

**Published:** 2024-06-10

**Authors:** Qi-ran Wang, Rong Yang

**Affiliations:** ^1^School of Education, Zhejiang International Studies University, Hangzhou, China; ^2^MuTri Doctoral School and Research Unit of Music Education, Jazz and Folk Music, Sibelius Academy, University of the Arts Helsinki, Helsinki, Finland

**Keywords:** MPA, career expectations, self-efficacy, early musical career, music students

## Abstract

Music performance anxiety (MPA) is recognized as a distinct emotional behavior rather than merely a motor control disorder and is influenced by specific conditioning experiences. This study investigates the interrelationships between MPA, self-efficacy, and future career expectations among music students within the Chinese context. The participants of this study were 340 high school students majoring in music education and performance, drawn from three music schools in China. Data were collected using several questionnaires: the MPA Inventory for Adolescents (MPAI-A), the Self-Efficacy Formative Questionnaire, and the Career Futures Inventory (CFI). The findings indicate that MPA is negatively associated with self-efficacy and future career expectations. Additionally, self-efficacy acts as a partial moderator between MPA and career expectations, suggesting that enhancing the self-efficacy of music students can boost their future career aspirations and mitigate the adverse effects of MPA. This research explores the complex relationships among MPA, self-efficacy, and future career expectations, emphasizing the importance of curriculum and pedagogical strategies in music schools. Music students with high levels of self-efficacy may exhibit more confidence and stable performances before audiences. According to the panel regression analysis, self-efficacy significantly positively influences career expectations. An appropriate educational environment and supportive pedagogical approaches to MPA can foster the early career development of musicians.

## 1 Introduction

Music performance anxiety (MPA) is recognized as a unique type of emotional behavior that extends beyond mere motor control disorders, originating from particular conditioning experiences and characterized by significant and persistent apprehension associated with musical performance (Kenny, 2012; Kesselring, 2012). The field of MPA research continues to expand within music education and psychology of music, presenting enduring challenges for music learners.

MPA affects not only professional musicians (Papageorgi and Welch, 2020; Butković et al., 2022) but also music students (Orejudo Hernández et al., 2018; Paliaukiene et al., 2018), adolescents (Thomas and Nettelbeck, 2014; Patston and Osborne, 2016), and children (Victoria Urruzola and Bernaras, 2020; Papageorgi, 2022). Its influence extends beyond musical aptitude, training, and levels of preparation to include intrinsic and extrinsic cognitive, as well as cultural factors (Salmon, 1990; Papageorgi et al., 2007). For instance, seasoned musicians may experience less anxiety arousal compared to inexperienced ones, who may peak during performances (Salmon et al., 1989; Dunkel et al., 1994; Ryan, 2004; Butzer et al., 2016). Furthermore, individual variables such as gender, experience, and weekly practice hours exhibit distinct correlations with MPA, especially when comparing early-career students to professional musicians (Biasutti and Concina, 2014). The manifestations of MPA include a variety of symptoms that appear through a combination of subjective feelings, cognitive, physical (Sabino et al., 2020), and behavioral changes (Kenny, 2009, 2012; Kesselring, 2012; Wiedemann et al., 2020). Individuals suffering from MPA experience persistent, distressful anxiety and impaired performance skills in public, which impact performance quality and leading to ongoing struggles with unrealistic expectations of failure and self-evaluation (Salmon, 1990; Kenny, 2009, 2012; Kesselring, 2012). Therefore, this research aims to enhance understanding of the relationship between MPA and related variables, thereby assisting educators, musicians, and researchers in developing more effective strategies to address these issues.

### 1.1 Music performance anxiety impairs musical career expectations

Given the adverse reactions to MPA, it is clear that MPA may impair the career expectations of musicians. However, research focusing on the correlation between MPA and the musical career expectations is limited. Orejudo Hernández et al. (2018) addressed that high levels of anxiety might lead early-career music students to consider abandoning their musical careers, thereby affecting their career expectations to MPA.

Career expectations is regarded as evaluation of positive attitudes toward future career planning, closely related to occupational designation of individuals, primarily encompassing career adaptability and optimism (Rottinghaus et al., 2005). Career adaptability, originating from Super’s career maturity theory, is particularly relevant for adults within the context of lifespan and life-space theory (Super, 1980, 1983). This concept denotes the ability to plan and modify career plans and work responsibilities in uncertain situations (Savickas, 1997). Career optimism involves anticipating future job-related outcomes, particularly as individuals who maintain a positive outlook on their career progression tend to remain confident despite challenges (Tolentino et al., 2014). Additionally, the correlation between career adaptability and career optimism can predict attitudes toward future career expectations across various age groups (Wilkins et al., 2014; Santilli et al., 2017; Delle and Searle, 2022).

Consequently, students suffering from high MPA might adapt to their musical career expectations with diminished career optimism. Conversely, individuals with strong career expectations are more likely to persist with their plans to achieve their goals. Developing career expectations has been shown to notably enhance career adaptability and foster career optimism. The interplay between career adaptability and optimism forms a virtuous cycle that could improve the career expectation of musicians, manage stress, and encourage the pursuit of careers despite the challenges posed by MPA. Therefore, integrating career expectations and MPA into a comprehensive stress management strategy may better prepare musicians and music students for more effective career planning.

### 1.2 Strengthening self-efficacy to manage MPA: a pathway to develop career expectations

To discover ways to alleviate and cope with MPA, numerous studies have investigated its correlates (Osborne et al., 2020; Wiedemann et al., 2020; Cohen and Bodner, 2021; Papageorgi, 2022), antecedents (Sabino et al., 2020; Butković et al., 2022), consequences (Kenny, 2009; MacAfee and Comeau, 2020), and coping strategies (Cohen and Bodner, 2019; Clarke et al., 2020; Huang and Song, 2021). Existing treatments for MPA are varied, ranging from the use of sedatives (Steptoe, 1989) and β-blockers (Brugués, 2011) to coping strategies such as self-monitoring with a ‘performance diary’ (PD) (Huang and Song, 2021), cognitive restructuring methods like arousal imagery (Finch et al., 2021), mindfulness strategies (Czajkowski et al., 2022) and emotional regulation (Kaleńska-Rodzaj, 2021).

Bandura (1977) defined self-efficacy as the belief in one’s ability to perform behaviors that lead to desired outcomes, influencing choices, efforts, and persistence in overcoming obstacles. Self-efficacy, a pivotal concept in social cognitive career theory (SCCT), is profoundly influenced by physiological arousal, such as anxiety. According to Bandura (1986), anxiety serves as one source of self-efficacy expectations, shaping one’s belief in their ability to successfully execute specific tasks. The relationship between music performance anxiety and self-efficacy is complex and multifaceted. While MPA can reduce self-efficacy by intensifying doubt and fear, leading to impaired performance, it also has the potential to enhance self-efficacy through successful management and channeling of anxiety in past performances. For example, studies have shown that improving self-efficacy, particularly in music students, can effectively reduce MPA (Liu, 2010). On the one hand, MPA negatively impacts self-efficacy by disrupting cognitive processing and diminishing concentration, which mediates its effects on performance and increases the likelihood of performance errors; on the other hand, a certain level of performance anxiety may be beneficial and even necessary for achieving peak performance quality (González et al., 2018; MacAfee and Comeau, 2020). Some level of MPA can heighten the arousal and focus of performers, leading to enhanced sensory perception and emotional engagement, crucial for expressive performances.

Investigating the relationships among MPA, self-efficacy, and career expectations is valuable and can be a strategy to support professional music education. Career expectation, as outcome expectations, are beliefs directly influenced by self-efficacy, shaping participants’ career interests, goals, and performance (Lent et al., 1994). Previous literature on self-efficacy and career expectations has primarily examined job satisfaction (Lent and Brown, 2006; Buyukgoze-Kavas et al., 2014) and sustainable job development (Liu et al., 2020; Kwee, 2021), with fewer studies focusing on preservice teachers or early-career students (Rogers et al., 2008; Li et al., 2021). McLennan et al. (2017) found that the self-efficacy of preservice teachers directly relates to their career optimism and moderates the effect of career adaptability, which is enhanced through the learning experience. Although researchers have reported on self-efficacy and MPA (Orejudo et al., 2017; Robson and Kenny, 2017; González et al., 2018; Paliaukiene et al., 2018; MacAfee and Comeau, 2020), its application in studies on music education and performance has been limited, often neglecting the importance of career development (Thornton and Bergee, 2008; Bulgren, 2017; Kuebel, 2019).

This research investigates the relationship among self-efficacy, career expectations as outcome expectations, and MPA as personal inputs in music students, positioning self-efficacy as a bridge between MPA and career expectations within the theoretical framework of SCCT. Music students, particularly those majoring in music performance or music education, constitute a unique group in the early stages of their musical careers. This research focuses on examining the impact of MPA on self-efficacy and future career expectations within Chinese cultural contexts, encompassing students from diverse musical backgrounds.

## 2 Materials and methods

### 2.1 Participants

The valid sample consisted of 340 participants from three music high schools in China. In terms of gender, 239 (70.3%) of the participants were female, and 101 (29.7%) were male. This gender distribution reflects the typical demographics of Chinese music students, where music schools often have a higher proportion of female students (Wang, 2016). The participants’ mean age was 16.12 years (SD = 0.91), with ages ranging from 15 to 19. The students were majoring in music education and music performance. Among those majoring in instrument performance, 122 were classical instrumentalists (piano, violin, clarinet, etc.), 41 were modern instrumentalists (electric guitar, bass guitar, jazz drum kit, etc.), and the remaining 66 were vocal performance students. A total of 111 participants were studying music education. The average years of performance experience among the participants was 7.5 years (SD = 0.817). Participants’ background information is summarized in Table 1.

**Table 1:** Sample and predictors descriptive statistics.

Gender		Age		Major		
Females	Males	M	SD	Instruments performance	Vocal performance	Music education
239	101	16.12	0.91	163	66	111
		M		SD	Min-Max	
MPA(K-MPA)		40.82		12.51	11–65	
Somatic and cognitive features		25.01		7.34	8–40	
Performance context		6.24		3.16	3–15	
Performance evaluation		9.57		3.61	2–17	
Student self-efficacy		47.29		11.38	31–64	
Belief in personal ability		29.12		6.79	18–40	
Belief that ability improves with effort		18.17		4.12	8–25	
Career futures inventory		78.32		17.67	48–112	
Career adaptability		35.21		7.81	21–55	
Career optimism		32.37		7.57	24–55	
Perceived knowledge of the job market		10.74		2.12	3–15	

### 2.2 Materials

#### 2.2.1 Music performance anxiety

This variable was assessed using the MPA Inventory for Adolescents (MPAI-A; Osborne Kenny and Holsomback, 2005), a self-report questionnaire comprising 15 items designed to evaluate the somatic, cognitive, and behavioral aspects of anxiety among adolescent musicians (e.g., Osborne Kenny and Holsomback, 2005; Thomas and Nettelbeck, 2014). The assessment is divided into three distinct categories: (1) somatic and cognitive features (8 items; e.g., During my performances, my hands tend to become sweaty.”); (2) performance context (3 items; e.g., I make an effort to refrain from playing alone at school.”); and (3) performance evaluation (4 items; e.g., Upon completing my performance, I typically feel satisfied with my presentation.”). Items are rated on a five-point Likert scale, with higher scores indicating higher levels of MPA. The internal consistency reliability coefficient for this scale in the present study was 0.760.

#### 2.2.2 Student self-efficacy

Student self-efficacy was assessed using the 13-item Self-Efficacy Formative Questionnaire (Gaumer Erickson and Noonan, 2018). In education, self-efficacy is linked to an individual’s belief in their capacity to achieve expected levels and accomplish predetermined goals or milestones. Self-efficacy essentially involves believing in one’s ability to tackle challenging tasks and to grow through effort (Gaumer Erickson and Noonan, 2016). The Self-Efficacy Formative Questionnaire is designed to evaluate a student’s perceived proficiency in two fundamental aspects of self-efficacy: belief in one’s personal ability (8 items; e.g., With daily practice, I could cultivate nearly any skill.”) and belief in one’s ability to meet or exceed goals and uphold expectations (5 items; e.g., I hold the belief that diligent effort yields positive outcomes.”). Each item is rated on a scale from 1 (very slightly or not at all) to 5 (extremely). The questionnaire results enable students to develop an understanding of their perceptions and beliefs about how their abilities contribute to their academic success. The internal consistency reliability for belief in personal ability was measured as 0.872, and for the belief that ability improves with effort, it was measured at 0.839.

#### 2.2.3 Career futures inventory

The Career Futures Inventory was assessed using 25 items developed by Rottinghaus et al. (2005), divided into three subscales. Career Adaptability: This subscale measures individuals’ perception of their capability to handle and capitalize on future changes, their comfort with new work responsibilities, and their ability to recover when unexpected events alter their career plans (11 items; e.g., I am good at adapting to new work settings.”). Career Optimism: This measures an individual’s disposition toward expecting the best possible outcome and emphasizing the most positive aspects of future career development and comfort in performing career planning tasks (11 items; e.g., I feel enthusiastic when contemplating my career.”). Perceived Knowledge of the Job Market: This subscale evaluates an individual’s understanding of job markets trends and employment dynamics (3 items; e.g., I am good at understanding job market trends”). Each measure is rated on a 5-point Likert scale from 1 (strongly disagree) to 5 (strongly agree). The responses were averaged, with high scores indicating elevated levels of outcome expectation. The Cronbach’s alpha coefficients were recorded as 0.925 for career adaptability, 0.896 for career optimism, and 0.869 for perceived knowledge of the job market.

### 2.3 Procedure

This study received the necessary authorizations from the Musicians’ Committees and School Management for student participation. The questionnaires, originally in English, were translated into Chinese by professional translators and then back-translated into English by another set of professionals. The back-translations were compared with the original version, and minimal changes were made until a consensus was reached. Participants completed the questionnaires in their preferred language; they were provided in two versions, counterbalanced for order.

Participation in this study was voluntary. All selected students were instructed to complete all questionnaires, which were filled out anonymously in classrooms arranged by the schools. Participants received specific instructions for each questionnaire and were informed that no question might touch on sensitive topics. Teachers and research assistants responsible for administering the questionnaires were trained in a standardized procedure for questionnaire administration. Participants spent approximately 15 min completing all questions, assigned online by an internal system. The study received approval from the music school and university’s ethical review committee. Upon completing the questionnaires, participants received a small gift in appreciation of their participation.

### 2.4 Statistical method

We analyzed the scores for MPA and self-efficacy, as well as participants’ perspectives on career expectations. To assess the influence of MPA on future career expectations, we conducted an ordinary least squares regression analysis of collected data. Additionally, using the moderation analysis model, we tested the moderating role of self-efficacy on the relationship between experiences of MPA and future career development.

## 3 Results

According to the results presented in Table 2, we observed that three facets of MPA—somatic and cognitive features anxiety, performance context anxiety, and performance evaluation anxiety—have a significant negative impact on career adaptability, with coefficients of −0.049, −0.061 and − 0.046, respectively, at the 0.01 level of significance. Furthermore, somatic and cognitive features anxiety and performance context anxiety significantly negatively affect individual career optimism and perceived knowledge, while performance evaluation anxiety does not significantly negatively impact career optimism and perceived knowledge. Overall, MPA negatively influences the career futures inventory of students.

**Table 2:** Effect of MPA on students’ career futures inventory.

Variables	Career adaptability (1)	Career optimism (2)	Perceived knowledge (3)
Somatic and cognitive features anxiety	−0.049***	−0.043***	−0.036***
	(0.008)	(0.008)	(0.010)
Performance context anxiety	−0.061***	−0.053***	−0.044**
	(0.016)	(0.015)	(0.018)
Performance evaluation anxiety	−0.046**	−0.029	−0.009
	(0.020)	(0.020)	(0.023)
Control variables	√	√	√
R-squared	0.433	0.335	0.181
adj. *R*^2^	0.421	0.322	0.169

*N* = 340; *p* < 0.05; **p < 0.01; ***p < 0.001; Control variables: Gender; Age, Major.

The results in Table 3, Panel A, reveal that self-efficacy has a significant negative impact on individual MPA. Specifically, for each unit increase in self-efficacy, reductions in individual somatic and cognitive features anxiety, performance context anxiety, and performance evaluation anxiety are observed at 2.270, 1.023, and 0.542 standard deviations, respectively. Additionally, self-efficacy has a significant positive effect on individual career adaptability, career optimism, and perceived knowledge, with influence factors of 0.377, 0.375, and 0.281, respectively, at the 0.01 level of significance.

**Table 3:** Effect of self-efficacy on MPA and the career futures inventory.

	Somatic and cognitive features anxiety	Performance context anxiety	Performance evaluation anxiety	Career adaptability	Career optimism	Perceived knowledge
Panel A	(1)	(2)	(3)	(4)	(5)	(6)
Self-efficacy	−2.270***	−1.023***	−0.542***	0.377***	0.375***	0.281***
	(0.474)	(0.224)	(0.203)	(0.065)	(0.062)	(0.068)
Control variables	√	√	√	√	√	√
Observations	340	340	340	340	340	340
R-squared	0.140	0.134	0.105	0.151	0.151	0.075
Panel B	(7)	(8)	(9)	(10)	(11)	(12)
Belief in personal ability	−3.798***	−1.287***	−1.314***	0.516***	0.384***	0.444***
	(0.526)	(0.304)	(0.264)	(0.071)	(0.072)	(0.079)
Belief that ability improves with effort	−0.438	−0.649**	−0.343	0.255***	0.306***	0.155**
	(0.540)	(0.267)	(0.236)	(0.071)	(0.074)	(0.077)
Control variables	√	√	√	√	√	√
R-squared	0.271	0.235	0.239	0.386	0.340	0.234
adj. *R*^2^	0.260	0.223	0.227	0.372	0.329	0.221

*N* = 340; *p < 0.05; **p < 0.01; ***p < 0.001; Control variables: Gender, Age, Major.

We also estimated the effects of belief in personal ability and the belief that ability improves with effort through self-efficacy on MPA and career development. The results, displayed in Panel B of Table 2, show that belief in personal ability significantly reduces the effects of somatic and cognitive features anxiety, performance context anxiety, and performance evaluation anxiety when students are performing music, with influence factors of −3.798, −1.287, and − 1.314, respectively, at the 0.01 level of significance. The belief that one’s ability improves with effort only has a significant negative effect only on performance context anxiety, with an influencing factor of −0.649 at the 0.05 level of significance. Career adaptability, career optimism, and perceived knowledge of the career futures inventory are significantly positively influenced by belief in personal ability and the belief that ability improves with effort.

To determine the moderating role of self-efficacy in MPA and the facets of the career futures inventory, a moderation model analysis was conducted. Models (1), (4), and (7) listed in Table 4 demonstrate the moderating role of self-efficacy in the effects of MPA on the career futures inventory of students. We found that when the interaction terms including self-efficacy were added, the negative effects of somatic and cognitive features anxiety and performance evaluation anxiety on career adaptability, career optimism, and perceived knowledge (the three factors of the career future inventory) were no longer significant, although the effects on career adaptability remained significantly negative.

**Table 4:** Moderating role of self-efficacy on MPA and the career futures inventory.

	Career adaptability			Career optimism			Perceived knowledge		
	(1)	(2)	(3)	(4)	(5)	(6)	(7)	(8)	(9)
Somatic and cognitive features anxiety	−0.041	−0.067**	−0.065*	−0.035	−0.016	−0.031	0.028	0.025	−0.015
	(0.031)	(0.032)	(0.033)	(0.027)	(0.029)	(0.034)	(0.034)	(0.035)	(0.044)
Performance context anxiety	−0.113*	−0.142**	−0.173**	−0.077	−0.160***	−0.192**	−0.070	−0.189***	−0.120
	(0.066)	(0.060)	(0.070)	(0.059)	(0.059)	(0.074)	(0.076)	(0.071)	(0.090)
Performance evaluation anxiety	−0.102	0.092	0.104	−0.096	0.057	0.106	−0.172*	0.056	0.015
	(0.080)	(0.065)	(0.081)	(0.075)	(0.071)	(0.088)	(0.088)	(0.084)	(0.105)
Self-efficacy	0.008			0.089			0.313		
	(0.144)			(0.142)			(0.190)		
Self-efficacy*Somatic and cognitive features anxiety	−0.001			−0.001			−0.017*		
	(0.009)			(0.008)			(0.010)		
Self-efficacy*Performance context anxiety	0.018			0.010			0.009		
	(0.019)			(0.018)			(0.023)		
Self-efficacy*Performance evaluation anxiety	0.016			0.019			0.048*		
	(0.022)			(0.021)			(0.026)		
Belief in personal ability		0.123			0.277*			0.425**	
		(0.147)			(0.151)			(0.206)	
Belief in personal ability*Somatic and cognitive features anxiety		0.009			−0.004			−0.013	
		(0.009)			(0.008)			(0.009)	
Belief in personal ability*Performance context anxiety		0.026			0.033**			0.044**	
		(0.017)			(0.016)			(0.019)	
Belief in personal ability*Performance evaluation anxiety		−0.034*			−0.021			−0.014	
		(0.018)			(0.020)			(0.023)	
Belief that ability improves with effort			0.073			0.190			0.214
			(0.153)			(0.172)			(0.212)
Belief that ability improves with effort# Somatic and cognitive features anxiety			0.006			−0.001			−0.004
			(0.009)			(0.009)			(0.011)
Belief that ability improves with effort# Performance context anxiety			0.034*			0.041**			0.024
			(0.018)			(0.019)			(0.023)
Belief that ability improves with effort# Performance evaluation anxiety			−0.037*			−0.033			−0.004
			(0.021)			(0.022)			(0.028)
Control variables	√	√	√	√	√	√	√	√	√
R-squared	0.465	0.526	0.516	0.378	0.416	0.438	0.213	0.278	0.241
adj. *R*^2^	0.453	0.512	0.502	0.344	0.407	0.425	0.201	0.257	0.224

*N* = 340; *p* < 0.05, **p < 0.01; ***p < 0.001; Control variables: Gender; Age, Major.

We also examined the moderating role of belief in personal ability on the effect of MPA on the career futures inventory of students. According to the results from Models (2), (5), and (8) shown in Table 4, when the interaction terms involving belief in personal ability and MPA were included, the negative effects of somatic and cognitive features anxiety on career adaptability remained significant, while the effects on career optimism and perceived knowledge were not significant. Additionally, the negative effects of performance context anxiety on the three factors of the career futures inventory were significant, whereas the effects on performance evaluation anxiety were not significant.

We further explored the moderating role of the belief that one’s ability improves with effort on the effect of MPA on the career futures inventory of students. According to the results from Models (3), (6), and (9) presented in Table 4, when the interaction terms involving the belief that ability improves with effort and MPA were added, the negative effects of somatic and cognitive features of anxiety on career adaptability remained significant, but the impacts on career optimism and perceived knowledge were no longer significant. The negative effects of MPA on career adaptability and career optimism continued to be significant; however, the effects on perceived knowledge were not significant. Furthermore, the effects of performance evaluation anxiety on career futures inventory were not significant.

## 4 Discussion

This research explores and tests the negative impact of MPA on self-efficacy and career expectations, with a specific focus on music students majoring in music education and performance. The results confirm that MPA had negative relationships with self-efficacy and career expectations. Additionally, self-efficacy acts as a moderating factor in the relationship between MPA and career expectations.

### 4.1 Primary findings

As supported by the literature review, numerous studies have addressed the implications of MPA, particularly its negative effects (Kenny, 2009; MacAfee and Comeau, 2020). A key finding of this study is that MPA directly negatively effects the career expectations of music students. This result is consistent with prior research, which indicates that high levels of anxiety may lead music students to consider abandoning their musical careers (Orejudo Hernández et al., 2018). High levels of MPA are associated with poor sleep quality, greater psychological distress, and increased alcohol consumption among musicians, adversely affecting not only their musical careers but also their overall health (Simoens et al., 2015). Although this study found no direct effect of performance evaluation anxiety on career optimism or perceived knowledge, this does not imply that performance evaluation anxiety has no influence on these variables. Indirect effects were observed on career optimism and perceived knowledge through the moderating role of self-efficacy.

This research reveals the association between self-efficacy and MPA, which contrasts with prior studies that identified weak or non-existent relationships between these two factors (Robson and Kenny, 2017; González et al., 2018; Paliaukiene et al., 2018; MacAfee and Comeau, 2020). Self-efficacy, often equated with confidence, can regulate the debilitating effects of music performance provided they have not become overly pronounced (Simoens et al., 2015). Thus, echoing findings from related research (González et al., 2018), music students with high levels of self-efficacy may demonstrate more confidence and stability in performance before audiences. Panel regression analysis shows that self-efficacy significantly positively effects career expectations, supporting earlier findings that self-efficacy correlates with the level of future career expectations (Kim, 2014; Michael, 2019).

Furthermore, self-efficacy acts as a partial moderator between MPA and career expectations, suggesting that enhancing music self-efficacy of music students can boost their future career aspirations and mitigate the adverse effects of MPA. This aligns with results from previous studies on the moderating role of self-efficacy (Orejudo et al., 2017), whereas lower self-efficacy may be associated with high MPA (Robson and Kenny, 2017; Paliaukiene et al., 2018). In this context, increasing self-efficacy may serve as a strategy to support managing MPA (Huang and Song, 2021). Schunk (1989) noted that while successes increase self-efficacy and failures diminish it, a well-established sense of efficacy remains relatively unaffected by occasional setbacks. Hence, even minor successes can positively influence an individual’s self-efficacy (Howard, 2019). Thus, students can develop self-efficacy by experiencing success.

### 4.2 Implications

This study has significant theoretical and practical implications. Theoretically, it contributes to educational and psychological research on MPA. It supports applicability of the Kenny MPA Inventory for Adolescents scale (MPAI-A; Osborne Kenny and Holsomback, 2005) across different cultural contexts, demonstrating its empirical utility and relevance particularly in an East Asian setting. Additionally, the study elucidates distinct relationships between MPA and self-efficacy, outlining specific pathways that influence career optimism.

Practically, the findings reveal that self-efficacy moderates the relationship between MPA and the future career expectations of music students. Students may develop strong performance abilities and positive career expectations based on their self-beliefs. This from of self-efficacy can be regarded as academic self-efficacy, where stronger self-efficacy is associated with higher expectations and positive attitudes toward one’s career and life (Lent et al., 1994; Kim, 2014).

Moreover, within the teaching and learning cycle, teachers play a crucial role in providing strategic guidance (Huang, 2019), such as supporting performance preparation, fcilitating communication, and setting realistic expectations for students (MacAfee and Comeau, 2022). Such support may help reduce MPA and enhance self-efficacy and career expectations. Furthermore, it is essential that teachers receive formal training in MPA management, which would enable them to effectively design curricula focused on performance preparation and anxiety management. Particularly in educational guidance, teachers can regularly assess capacity and self-efficacy to identify self-efficacy levels of students. This assessment serves as an indicator of psychological states of students, enabling tailored support for each individual.

Addressing MPA, enhancing self-efficacy, and fostering career expectations are critical issues for both students and teachers. Additionally, school leadership should consider the importance of MPA, self-efficacy, and career expectations, not merely focusing on career-oriented curricula but also on developing skills and knowledge relevant for students who will become musicians, educators, or researchers. It is imperative to find effective ways to positively influence self-efficacy and career planning attitudes.

### 4.3 Limitations and future research

In this study, data were collected from participants’ self-reported responses without knowledge of their previous histories or how their views might have changed over time. Furthermore, our participants were enrolled in music schools, which may have standards and conditions that differ from other educational institutions in China. Future research should include music students from other schools or universities across China to broaden the findings.

Further research could build upon our findings and the relationships identified here. Newly developed models should consider all emerging variables to deepen the understanding of the dynamics at play. It would be particularly insightful to explore not just if, but when and how music performance and self-efficacy influence each other. Future studies could also examine the preservice teacher preparation programs, such as those utilizing the Alexander Technique, to determine more effective ways of coping with MPA.

## Data availability statement

The raw data supporting the conclusions of this article will be made available by the authors, without undue reservation.

## Ethics statement

Ethical review and approval was not required for the study on human participants in accordance with the local legislation and institutional requirements. Written informed consent from the patients/participants or patients/participants legal guardian/next of kin was not required to participate in this study in accordance with the national legislation and the institutional requirements.

## Author contributions

Q-rW: Writing – review & editing, Writing – original draft, Resources, Project administration, Methodology, Investigation, Funding acquisition, Formal analysis, Data curation, Conceptualization. RY: Writing – review & editing, Writing – original draft, Resources, Project administration, Methodology, Investigation, Data curation, Conceptualization.
